# PRELIMINARY FINDINGS OF A STUDY ON LONGITUDINAL CHANGES IN EXPRESSED EMOTION IN FAMILY CAREGIVERS WITH DEMENTIA

**DOI:** 10.1093/geroni/igac059.3111

**Published:** 2022-12-20

**Authors:** Xiaoji Liu, Reiko Kanaya, Kazue Shigenobu, Keigo Takiue, Miyae Yamakawa

**Affiliations:** Osaka University, suita, Osaka, Japan; Osaka University, suita, Osaka, Japan; Osaka University, suita, Osaka, Japan; Gifu University, Gifu, Gifu, Japan; Osaka University, suita, Osaka, Japan

## Abstract

**Purpose:**

The aim of this study was to explore and describe changes in expressed emotion (EE) among family caregivers of dementia and its associated factors at different time points over 3 months. Method: A longitudinal observation study using a 3-month survey was conducted in home care settings, Japan. We collected data on the demographic details of family caregivers currently providing care, which included care burden (Zarit Burden Inventory, ZBI), closeness of the caregiving relationship (Relationship Closeness Scale, RCS), depression (Geriatric Depression Scale, GDS). We used the validated Family Attitude Scale (FAS) to assess EE, in which higher scores indicate a greater intensity of emotional expression.

**Results:**

To date, 45 family caregivers completed the present study. FAS scores were 39.67 ± 24.90 at month 1 (T1), 38.64 ± 23.28 at month 2 (T2), and 39.69 ± 24.52 at month 3 (T3). The change in FAS scores did not show a statistical difference over the three-month period (p=0.816). Multiple regression analysis of FAS at T1 showed that the main influencing factors were gender, marriage, RCS, care burden, and depression (R2=0.763, p < 0.001); the main influencing factors at T2 were marriage and care burden (R2=0.584, p < 0.001); the main influencing factors at T3 were care burden and depression (R2=0.674, p < 0.001). Conclusions: Early identification of risk factors may help in the development of interventions to prevent high levels of EE in family caregivers. We plan to continue refining our analyses to test the validity of our hypothesis.

